# Arterial Spin Labeling Magnetic Resonance Imaging Can Identify Posterior Fossa Hemangioblastoma: Comparison with Dynamic Susceptibility Contrast

**DOI:** 10.3390/cancers18121926

**Published:** 2026-06-12

**Authors:** Takeshi Hiu, Ayano Ishiyama, Minoru Morikawa, Shimpei Morimoto, Ayaka Matsuo, Hikaru Nakamura, Hirofumi Koike, Yaojing Lin, Shiro Baba, Kenta Ujifuku, Koichi Yoshida, Ryo Toya, Takayuki Matsuo

**Affiliations:** 1 Department of Neurosurgery, Nagasaki University Graduate School of Biomedical Sciences, Nagasaki 852-8501, Japan; heygoo25@live.jp (A.M.); hikaru.nakamura560@gmail.com (H.N.); yaojing.lin@outlook.com (Y.L.); shiro.baba@nagasaki-u.ac.jp (S.B.); kentaujifuku@nagasaki-u.ac.jp (K.U.); kou-yoshida@nagasaki-u.ac.jp (K.Y.); takayuki@nagasaki-u.ac.jp (T.M.); 2 Department of Radiology, Nagasaki University Hospital, Nagasaki 852-8501, Japan; ahno@nagasaki-u.ac.jp (A.I.); m-minoru@nagasaki-u.ac.jp (M.M.); hkoike@nagasaki-u.ac.jp (H.K.); toya@nagasaki-u.ac.jp (R.T.); 3 Innovation Platform & Office for Precision Medicine, Nagasaki University Graduate School of Biomedical Sciences, Nagasaki 852-8523, Japan; 4 Clinical Research Center, Nagasaki University Hospital, Nagasaki 852-8501, Japan

**Keywords:** arterial spin labeling, cerebellar tumor, dynamic susceptibility contrast, hemangioblastoma, magnetic resonance imaging, posterior fossa tumor

## Abstract

Background/Objectives: Diagnosing hemangioblastomas using magnetic resonance imaging (MRI) is challenging, especially when the tumors appear as solid posterior fossa masses. This study aimed to evaluate the diagnostic performance of perfusion MRI and identify the most useful quantitative features for differentiating hemangioblastomas from other posterior fossa tumors. Methods: Forty-five posterior fossa tumors were analyzed, including 18 hemangioblastomas (HB group) and 27 non-hemangioblastoma tumors (NHB group; 8 metastatic brain tumors, 6 pilocytic astrocytomas, 5 malignant lymphomas, 4 glioblastomas, 2 medulloblastomas, and 2 other tumors). All patients underwent 3.0-T MRI. Arterial spin labeling (ASL) was used to calculate the relative tumor blood flow normalized to the contralateral gray matter. Dynamic susceptibility contrast (DSC) imaging was used to obtain regional cerebral blood flow, regional and corrected cerebral blood volume (CBV), and permeability index (K2) values. Regions of interest (ROIs) were placed within the contrast-enhancing areas. Results: The relative ASL values and corrected CBV were significantly higher in hemangioblastomas than in other tumors (*p* < 0.001). Relative ASL showed the highest diagnostic performance (sensitivity, 100%; specificity, 93.3%). Conclusions: Non-contrast ASL showed strong diagnostic performance for identifying posterior fossa hemangioblastomas and may serve as a practical alternative to contrast-enhanced DSC, although ROI placement can be challenging in very small mural nodules.

## Simple Summary

Hemangioblastomas are benign World Health Organization grade I vascular central nervous system tumors that arise sporadically or as manifestations of von Hippel–Lindau disease. This study investigated the utility of arterial spin labeling, a noninvasive magnetic resonance imaging technique, compared with dynamic susceptibility contrast imaging-derived metrics for differentiating hemangioblastomas from other types of posterior fossa brain tumors. Across two adult patient cohorts, hemangioblastomas consistently showed much higher blood flow than non-hemangioblastoma tumors. Among several imaging measures, including regional cerebral blood flow, regional and corrected cerebral blood volume, and permeability index values, arterial spin labeling-derived perfusion showed the strongest diagnostic performance, with very high sensitivity and specificity. Arterial spin labeling imaging may help identify highly vascular tumors, such as hemangioblastomas, without the need for contrast agents, making it a useful and noninvasive tool for improving diagnosis and supporting clinical decision-making in patients with posterior fossa brain tumors.

## 1. Introduction

Hemangioblastomas are benign World Health Organization grade I vascular central nervous system tumors that arise sporadically or as manifestations of von Hippel–Lindau disease. The overall incidence of central nervous system hemangioblastomas is low, with data from the United States indicating an overall incidence rate of 0.141 per 100,000 person-years [1]. Hemangioblastomas account for approximately 1–2.5% of intracranial tumors [1,2] and 7–12% of posterior fossa tumors [2]. They are most commonly found in the cerebellum (45–50%), followed by the spinal cord (40–45%) and brainstem (5–10%) [3,4,5].

From a histological perspective, hemangioblastomas consist of stromal cells with plump foamy cytoplasm and profuse capillary networks [6], with subclassification into reticular and cellular variants [6,7]. Common magnetic resonance imaging (MRI) findings in cases of hemangioblastoma include small, vascular, intensely contrast-enhancing mural nodules associated with an adjacent non-contrast-enhancing cystic mass [8]. However, a substantial proportion of hemangioblastomas presents as solid tumors [8], making accurate diagnosis difficult, particularly in the posterior fossa where multiple tumor types share similar imaging characteristics [9]. Recent deep-learning approaches based on contrast-enhanced T1-weighted imaging have also been explored to improve preoperative discrimination of hemangioblastoma from other posterior fossa tumors [10].

Hemangioblastomas are characterized by marked vascularity and dense tumor staining on angiography. A number of techniques have been devised for evaluating cerebral perfusion imaging [11,12,13], of which the dynamic susceptibility contrast (DSC) method is the most widely employed among clinical MR imaging professionals [14]. DSC imaging methods exploit signal changes accompanying the passage of a paramagnetic contrast agent through the cerebrovascular system, with this information used to derive data on blood volume and flow [15,16,17,18,19]. Studies using DSC and dynamic contrast-enhanced MR perfusion imaging have reported increased vascular perfusion in hemangioblastomas [20], relative to brain metastases [21,22], pilocytic astrocytomas [20,22], and medulloblastomas [22,23]. Arterial spin labeling (ASL) is a noninvasive MR perfusion technique that uses magnetically labeled arterial water instead of exogenous contrast agents, and it has been shown in a recent systematic review and meta-analysis to offer potentially comparable, non-inferior diagnostic accuracy to DSC in pediatric patients with a range of high- and low-grade brain tumors [24]. Despite these findings, relatively little is known regarding the comparative diagnostic performance of DSC versus ASL in adult patients with hemangioblastoma. This study aimed to explore the use of MRI-based perfusion imaging to diagnose hemangioblastomas and to identify the most informative quantitative features, specifically comparing DSC and ASL. This study extends prior work by systematically comparing ASL-derived perfusion and DSC-based metrics in posterior fossa tumors.

## 2. Materials and Methods

### 2.1. Patients

We reviewed the clinical and imaging records and surgical pathology results of patients with posterior fossa tumors at our institution between January 2013 and August 2021. All diagnoses were confirmed by histopathological examination of the surgical specimens, and patients without histopathological confirmation were excluded. In the first cohort (cohort 1), patients were included if they had (1) an intra-axial posterior fossa tumor and (2) both ASL and DSC imaging findings. A total of 27 patients with confirmed pathological tumor diagnoses were stratified as follows: HB group (12 hemangioblastomas) and NHB group (15 non-hemangioblastomas: 6 metastatic brain tumors, 3 pilocytic astrocytomas, 3 malignant lymphomas, 2 glioblastomas, and 1 medulloblastoma).

In the second cohort (cohort 2), patients were included if they had (1) an intra-axial posterior fossa tumor and (2) ASL imaging. Forty-five tumors met the inclusion criteria as follows: HB group (18 hemangioblastomas) and NHB group (27 non-hemangioblastomas: eight metastatic brain tumors, six pilocytic astrocytomas, five malignant lymphomas, four glioblastomas, two medulloblastomas, and two others).

Pediatric patients were defined as those aged <18 years. In the first cohort, NHB group included 2 pediatric patients; in the second cohort, NHB group included 7 pediatric patients.

The second cohort represents an expanded cohort of patients who underwent ASL during the same study period. Therefore, the second cohort includes all patients from the first cohort, as the first cohort is the subset in which both ASL and DSC were available. A prediction model (see Section 2.5) was developed using the first cohort (development cohort; HB, *n* = 12; NHB, *n* = 15), which was evaluated by predictive performances on a subset of the second cohort after excluding all patients from the first cohort (validation cohort; HB, *n* = 6; NHB, *n* = 12).

### 2.2. MRI Protocol

All MRI images were obtained using a 3.0-T unit (Sigma HDxt; GE Healthcare, Milwaukee, WI, USA) with an 8-channel phased-array head coil. ASL, and DSC perfusion images were obtained during the same session.

Three-dimensional ASL was performed using a pseudo-continuous labeling period of 1500 ms, followed by a 2000 ms post-label delay. Whole axial brain images were obtained using a three-dimensional (3D) background-suppressed fast spin-echo stack-of-spirals method, with a repetition time (TR)/echo time (TE) of approximately 5238/13 ms and field of view (FOV) of 24 × 24 cm. Multi-arm spiral imaging was used, with four arms and 1024 points acquired on each arm (reconstruction matrix size, 128 × 128); slice thickness/gap = 6 mm/0 mm; and number of sections = 30.

DSC was performed using single-shot spin-echo echo-planar imaging (SE-EPI) with the following parameters: TR/TE = 1500/104 ms; FOV = 28 × 28 cm; number of excitations = 1; matrix size = 128 × 128; and 1 s intervals for 90 s. A bolus injection of gadolinium-based contrast medium (Magnevist^®^, Bayer HealthCare Pharmaceuticals, Berlin, Germany) at an injection rate of 3 mL/s, followed by a 20 mL saline flush, was performed for DSC acquisition and subsequent T1-weighted post-contrast imaging.

### 2.3. Image Analysis

All perfusion datasets provided full tumor coverage based on contrast-enhanced T1-weighted imaging. DSC data were processed using an Olea Sphere V3.0 (Olea Medical, Vitrea Workstation V7.1, Canon Medical Systems, Ōtawara, Japan). Region of interest (ROI) placement was performed manually. Two readers (T.H. and A.I.), who were blinded to the final histopathological diagnosis, independently delineated ROIs on contrast-enhanced T1-weighted images. For each tumor, measurements were performed on the axial slice demonstrating the largest solid enhancing component. ROIs were drawn to encompass the solid enhancing portion as much as possible while avoiding necrotic/cystic areas and macrovessels. The mean of the two measurements was used for analysis. ROI placement may be challenging in lesions with very small enhancing mural nodules, potentially leading to partial-volume effects; this limitation is addressed in the Discussion section. The regional cerebral blood volume (rCBV) values were calculated using block-circulant singular-value decomposition with leakage correction, and the permeability index (K2) values were computed using the method reported by Boxerman et al. [25]. Additionally, regional cerebral blood flow (rCBF) was obtained from the DSC perfusion analysis and is reported in mL/100 mL/min. Corrected rCBV was treated as a unitless relative measure.

For ASL, the relative tumor blood flow (rTBF) was calculated by normalizing the absolute tumor blood flow to that of the cortical gray matter in the contralateral hemisphere based on established methods. For tumors crossing the midline, the reference ROI was placed in the hemisphere least affected by the lesions.

### 2.4. Statistical Analysis

Analyses were performed using IBM SPSS (version 25.0; IBM Corp., Armonk, NY, USA), JMP 14.2 (SAS Institute, Cary, NC, USA), and R version 4.4.1 (R Foundation for Statistical Computing, Vienna, Austria). Data distribution was assessed using the Shapiro–Wilk test. Continuous variables were compared between groups using the Mann–Whitney U test, and categorical variables were compared using the chi-square test or Fisher’s exact test, as appropriate.

Receiver operating characteristic (ROC) analyses were performed, and areas under the ROC curve (AUCs) are reported with 95% confidence intervals (CIs). AUCs were compared using the paired DeLong test. Sensitivity and specificity are reported with 95% CIs calculated using the exact binomial (Clopper–Pearson) method. Cutoff values for corrected rCBV and rCBF were selected by maximizing the Youden index in cohort 1, whereas the relative ASL cutoff was fixed at 2.3 for the primary and validation analyses. Internal validation was performed using bootstrap resampling (B = 2000) to obtain optimism-corrected AUC estimates.

For the model-comparison analysis in cohort 1, we compared conventional MRI morphology (MRI-only), ASL-only, combined MRI + ASL, and DSC-based models; discrimination was summarized by AUC (95% CI), and AUCs were compared using the paired DeLong test with internal validation by bootstrap resampling (B = 2000) (Supplementary Table S1). The methods used in the development and evaluation of the predictions are detailed in Text S1, aligning with the TRIPOD + AI reporting guidelines [26].

### 2.5. Ethics Statements

This study was approved by the Ethics Committee of Nagasaki University Hospital (approval number 22022112-4). This was a retrospective study of medical records, and the data were analyzed anonymously. All the clinical investigations described in this study were conducted in accordance with the principles of the Declaration of Helsinki.

## 3. Results

In total, 27 lesions in 27 patients were evaluated in the first cohort, comprising 12 patients with hemangioblastomas (HB group) and 15 patients without hemangioblastomas (NHB group) (Supplementary Figures S1 and S2). The clinical and demographic information of the patients is presented in Table 1. Of these, 14 (51.9%) were men and 13 (48.1%) were women, with a median age of 57.0 years. Patients in the NHB group were older, with a median age of 63.0 years (range, 24.0–67.0), compared with a median age of 43.0 years (range, 37.0–50.3) in the HB group. Male patients accounted for 53.3% (8/15) and 50% (6/12) of the patients in the NHB and HB groups, respectively.

Lesions most commonly involved the cerebellar hemisphere in both groups, occurring in 53.3% (8/15) of the patients in the NHB group and in 66.7% (8/12) of the patients in the HB group. Vermian or midline involvement was observed in 26.7% (4/15) and 16.7% (2/12) of the NHB and HB groups, respectively, whereas cerebellar peduncle involvement was present in 20.0% (3/15) and 16.7% (2/12) of the patients, respectively. Multiple lesions were identified in 27.0% (4/15) of the NHB group but were not observed in the HB group.

Regarding lesion morphology, predominantly (>80%) solid lesions were more frequent in the NHB group, occurring in 66.7% (10/15) of the patients, compared with 25.0% (3/12) in the HB group. Mixed solid and cystic morphologies were observed in 6.7% (1/15) and 25.0% (3/12) of patients in the NHB and HB groups, respectively. Cystic lesions (>80%) with an enhancing mural nodule were identified in 20.0% (3/15) of the patients in the NHB group and 50.0% (6/12) of the patients in the HB group. Necrotic lesions with irregular walls were observed in 6.7% (1/15) of the patients in the NHB group but were not present in the HB group.

Median lesion volume was 16.4 mL (range, 12.3–30.6) in the NHB group and 13.5 mL (range, 7.3–21.1) in the HB group. Extratumoral cysts were more common in the HB group, occurring in 50.0% (6/12) of patients, compared with 6.7% (1/15) in the NHB group. Intratumoral cysts were observed in 33.3% (5/15) and 41.7% (5/12) of the NHB and HB groups, respectively, whereas solid lesions without cystic components were present in 60.0% (9/15) and 8.3% (1/12) of the NHB and HB groups, respectively.

Hydrocephalus was present in 40.0% (6/15) of the patients in the NHB group and 50.0% (6/12) of the patients in the HB group. Homogeneous contrast enhancement was observed in 27.0% (4/15) and 67.0% (8/12) of the patients in the NHB and HB groups, respectively.

**Figure 1:**
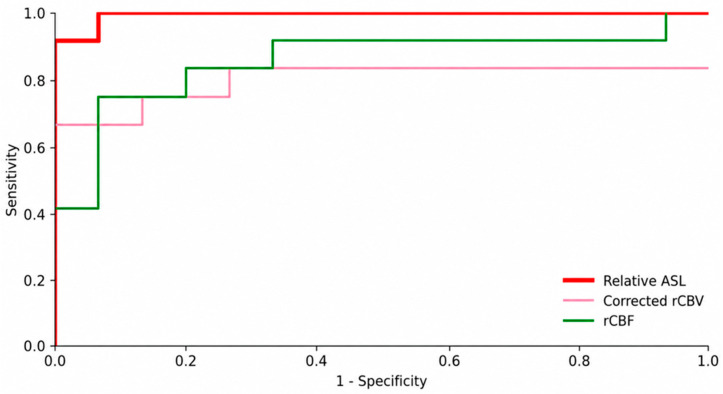
ROC analysis for the first cohort.

Table 2 shows the various MRI parameters in the first cohort. The relative ASL values in the HB group, as well as the corrected rCBV and rCBF values on DSC imaging, were significantly higher than those in the NHB group (*p* < 0.001). Receiver operating characteristic (ROC) analysis (Figure 1) demonstrated that the area under the ROC curve (AUC) for the relative ASL value was 0.994, which was higher than that for any other perfusion parameter (cutoff value, 2.3; sensitivity, 100%; specificity, 93.3%; Table 3). In a sensitivity analysis excluding the two pediatric patients (<18 years) in the first cohort, the diagnostic performance of relative ASL remained essentially unchanged (AUC, 0.994; sensitivity, 100%; specificity, 92.3%) using the fixed cutoff of 2.3.

Supplementary Table S1 summarizes the results of discrimination analyses comparing conventional MRI morphology, ASL, combined MRI + ASL, and DSC-based models with internal validation. ASL-only demonstrated superior discrimination compared with MRI morphology alone (AUC, 0.994 versus 0.786), whereas combining MRI morphology and ASL yielded the highest performance (AUC, 1.000). Bootstrap internal validation using the Harrell optimism correction demonstrated sustained model performance after correction for optimism.

**Figure 2:**
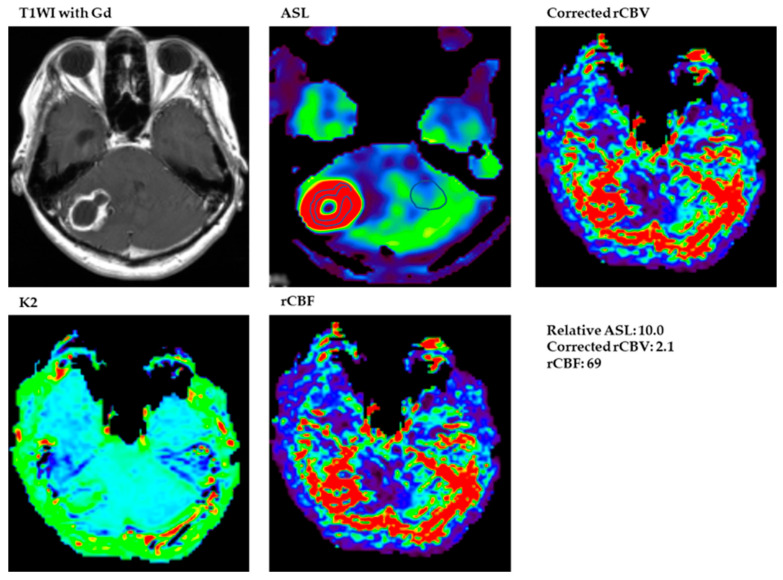
Representative case of hemangioblastoma with marked hyperperfusion on ASL. Representative images from a 52-year-old woman with a posterior fossa hemangioblastoma (HB group). Top row, left to right: Axial T1-weighted MRI after gadolinium administration, ASL map, corrected rCBV map, and perfusion graph. Bottom row, left to right: K2 and rCBF maps. The relative ASL value was 10.0, with a corrected rCBV of 2.1 and an rCBF of 69. Quantitative values are reported using ROI-based measurements. Color scales are provided in Supplementary Figure S2.

**Figure 3:**
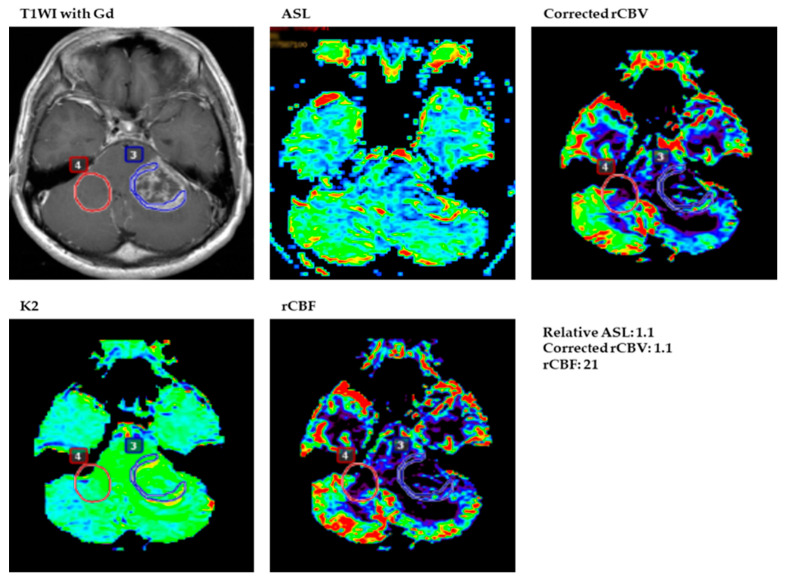
Representative case of glioblastoma showing low perfusion on ASL compared with hemangioblastoma. A representative image of a 24-year-old man with glioblastoma (NHB group). Top row, left to right: Axial T1-weighted MRI after gadolinium administration, ASL map, corrected rCBV map, and perfusion graph. Bottom row, left to right: K2 and rCBF maps. The relative ASL value was 1.1, with a corrected rCBV of 1.1 and rCBF of 21.

Figure 2 and Figure 3 show representative images from two patients in the first study cohort, one from the HB group and one from the NHB group. Figure 2 shows images acquired from a 52-year-old woman with a posterior fossa hemangioblastoma (HB group). The representative images include contrast-enhanced T1-weighted images and perfusion maps (ASL, corrected rCBV, rCBF, and K2). Axial T1-weighted MRI after gadolinium administration revealed an enhancing lesion in the right cerebellar hemisphere. ASL imaging demonstrated high signal intensity in the same anatomical region that showed enhancement on post-gadolinium T1-weighted imaging. More diffuse increases in the corrected rCBV and rCBF were observed on axial imaging. The relative ASL value was 10.0, with a corrected rCBV of 2.1 and an rCBF of 69 mL/100 mL/min.

Figure 3 shows the images acquired from a 24-year-old man with glioblastoma (NHB group). The representative images include contrast-enhanced T1-weighted imaging and perfusion maps. Axial T1-weighted MRI after gadolinium administration revealed an enhancing lesion in the left cerebellar hemisphere. ASL imaging demonstrated low signal intensity in the same anatomical region that showed enhancement on post-gadolinium administration in T1-weighted imaging, indicating low perfusion. Low corrected rCBV and rCBF were observed on axial imaging. The relative ASL value was 1.1, with a corrected rCBV of 1.1 and rCBF of 21 mL/100 mL/min.

In the second cohort, the relative ASL value in the HB group was significantly higher than that in the NHB group (*p* < 0.001; Table 4). The HB group showed an elevated median relative ASL value (median, 9.5) compared with the NHB group (median 1.2; Table 5). Using the fixed cutoff value of 2.3 derived in the first cohort, diagnostic performance was evaluated in the second cohort; because the cohort 2 includes cohort 1, we additionally assessed performance in the non-overlapping subset (Table 6). The distribution of relative ASL values across NHB pathological subtypes is shown in Supplementary Figure S3.

## 4. Discussion

Perfusion MRI has been widely used to noninvasively evaluate tumor vascularity and angiogenesis and shows good correlation with histological assessments. The current study evaluated the imaging and clinical characteristics of patients with posterior fossa intra-axial tumors across two cohorts, comparing patients with hemangioblastomas (HB group) to patients without hemangioblastomas (NHB group) [15,27]. The main focus of this study was to investigate the diagnostic value of ASL imaging for differentiating hemangioblastomas from other posterior fossa tumors. In the first cohort of 27 patients who underwent both ASL and DSC imaging, marked differences in perfusion imaging results were observed between the two groups. The HB lesions demonstrated significantly higher perfusion across multiple parameters, including ASL, corrected rCBV, and rCBF (all *p* < 0.001). Among these, relative ASL was the most discriminative parameter, with excellent diagnostic performance (AUC 0.994), achieving 100% sensitivity and 93.3% specificity at a cutoff of 2.3. Because the second cohort includes the first cohort, it does not represent an independent validation cohort; therefore, we additionally assessed the fixed cutoff in the non-overlapping subset of the second cohort (Table 6). This supports the potential role of ASL as a non-contrast perfusion technique for evaluating posterior fossa tumors. Relative ASL showed higher discriminative ability than DSC-derived parameters for distinguishing hemangioblastomas from non-hemangioblastomas; however, the DeLong comparison between relative ASL and corrected rCBV did not reach statistical significance. Bootstrap internal validation supported the robustness of ASL diagnostic performance after optimism correction.

Relative tumor blood flow measured by ASL is significantly higher in hemangioblastomas than in metastatic tumors [28,29]. In contrast, our study included a larger sample than previous ASL studies and directly compared ASL with DSC perfusion metrics, enabling a more robust assessment of their diagnostic performance. ASL is a promising perfusion MR method because it is repeatable, cost-effective, and entirely noninvasive, as it does not require the administration of exogenous contrast agents [30]. These characteristics are especially advantageous in patients with renal dysfunction, allergy to contrast media, or when repeated follow-up imaging is required. The usefulness of ASL in evaluating and differentiating brain tumors has been demonstrated [31,32], and our findings further support its value in the posterior fossa, where anatomical complexity often complicates diagnosis. Notably, a characteristic qualitative ASL pattern (“lightbulb sign”), characterized by intense and homogeneous hyperperfusion within the solid component, has recently been described in posterior fossa hemangioblastoma and aligns with the marked hyperperfusion observed in our hemangioblastoma cases [33].

In particular, the posterior fossa presents unique imaging challenges owing to beam-hardening artifacts, susceptibility effects from the adjacent bone, and the compact arrangement of critical neurovascular structures. In this context, ASL provides an alternative to conventional perfusion techniques by offering hemodynamic information without the susceptibility-related limitations that may affect DSC imaging [30].

In addition to its technical advantages, ASL offers important physiological insights into tumor vascularity and perfusion characteristics. Tumor blood flow, as measured by ASL, reflects the underlying angiogenesis and microvascular density, which are key determinants of tumor behavior and aggressiveness. Prior investigations have demonstrated the utility of ASL in differentiating tumor recurrence from treatment-related changes such as radiation necrosis, highlighting its role not only in initial diagnosis but also in longitudinal disease monitoring [29]. Furthermore, ASL-derived perfusion metrics have shown good correlation with established perfusion parameters, such as relative CBV obtained from DSC imaging, supporting its validity as a quantitative imaging biomarker [28]. In the present study, the marked differences in ASL perfusion values between hemangioblastomas and non-hemangioblastoma lesions reinforce the sensitivity of ASL to variations in tumor vascularity, particularly in highly vascular tumors.

Importantly, ASL may also contribute to improved clinical decision-making by enhancing diagnostic confidence and potentially reducing the need for invasive procedures or contrast-enhanced studies. Its repeatability makes it well-suited for treatment response assessment and surveillance, particularly in patients requiring frequent imaging follow-up. However, it should be acknowledged that ASL is not without limitations, including sensitivity to arterial transit time and a lower signal-to-noise ratio compared with DSC techniques. Despite these challenges, technical refinement continues to improve image quality and quantitative accuracy. Taken together, our current findings and those presented in the prior literature suggest that ASL represents a valuable adjunct to conventional MRI, particularly in complex anatomical regions such as the posterior fossa, and may play an increasingly important role in the comprehensive evaluation of posterior fossa tumors. In parallel, transfer-learning and deep-learning approaches have been applied to brain MRI classification tasks, suggesting potential for automated decision support; integrating physiologically interpretable perfusion biomarkers such as ASL may improve robustness and clinical utility of such models [10,34].

A limitation of our study includes the retrospective design. Additional prospective studies in larger populations are required to further explore the potential advantages of ASL imaging in the challenging clinical area of posterior fossa tumor diagnosis. Other limitations include the single-center design, which may have introduced selection bias, and the relatively small sample size, particularly in the cohort in which both ASL and DSC data were available. In addition, ROI-based measurements are operator dependent, and interobserver and intraobserver variability may affect the reproducibility of perfusion metrics. Accurate ROI placement can be challenging in lesions with very small enhancing mural nodules, potentially leading to partial-volume effects and reduced measurement reliability. Although ROIs were independently delineated by two readers and averaged, formal reproducibility metrics (e.g., intraclass correlation coefficients) were not calculated and should be evaluated in future studies. Furthermore, imaging was performed on a single 3.0-T scanner using a specific ASL/DSC acquisition protocol and post-processing pipeline. Therefore, the generalizability of the proposed cutoff values to other scanners, sequences, and software platforms requires further validation. Finally, not all participants underwent DSC imaging. To address this, we analyzed two cohorts, but direct ASL–DSC comparisons were limited to the first cohort. As the second cohort included the first cohort, it did not constitute an independent external validation cohort; although we assessed performance in a non-overlapping subset, independent multi-center validation remains necessary. Given the small sample size, multivariable modeling may be unstable and should be interpreted cautiously despite internal validation. Prospective multicenter studies with standardized acquisition/analysis and reproducibility testing are warranted.

## 5. Conclusions

Non-contrast ASL showed strong diagnostic performance for identifying hemangioblastomas among posterior fossa tumors. ASL may also be valuable in postoperative surveillance, as it has been reported to assist in detecting residual or recurrent tumors. Given its strong diagnostic performance, ASL may serve as a practical alternative to contrast-enhanced DSC in selected settings, suggesting that gadolinium-based perfusion imaging may not always be essential for evaluating posterior fossa hemangioblastomas. However, accurate ROI placement may be challenging in cases where the mural nodule is very small, which should be considered when interpreting ASL findings.

## Figures and Tables

**Table 1:** Clinical and demographic characteristics of patients in the first cohort.

	Hemangioblastoma (HB Group)	Non-Hemangioblastoma (NHB Group)
N	12	15
Age, years (median, range)	43.0 (37.0–50.3)	63.0 (24.0–67.0)
Gender (male)	6 (50%)	8 (53.3%)
Location		
Cerebellar hemisphere	8 (66.7%)	8 (53.3%)
Vermis/midline	2 (16.7%)	4 (26.7%)
Cerebellar peduncle involvement	2 (16.7%)	3 (20.0%)
Multiple	0 (0.0%)	4 (27.0%)
Lesion morphology		
Predominantly (>80%) solid	3 (25.0%)	10 (66.7%)
Mixed solid and cystic	3 (25.0%)	1 (6.7%)
Cystic (>80%) with enhanced nodule	6 (50.0%)	3 (20.0%)
Necrotic with irregular wall	0 (0.0%)	1 (6.7%)
Volume (mL)	13.5 (7.3–21.1)	16.4 (12.3–30.6)
Cyst type		
Extratumoral cyst	6 (50.0%)	1 (6.7%)
Intratumoral cyst	5 (41.7%)	5 (33.3%)
Solid	1 (8.3%)	9 (60.0%)
Hydrocephalus	6 (50.0%)	6 (40.0%)
Enhancement pattern: homogeneous	8 (67.0%)	4 (27.0%)

**Table 2:** MRI parameter data in the first cohort.

	HB Group	NHB Group	*p* Value
Relative ASL median (25–75th percentile)	9.6 (5.2 to 11.6)	1.2 (0.7 to 1.5)	<0.001
DSC perfusion			
Corrected rCBV	6.3 (2.0 to 7.2)	1.0 (0.7 to 1.8)	0.001
rCBV	6.7 (1.7 to 8.8)	1.1 (0.4 to 2.1)	0.085
rCBF	84 (65 to 112)	30 (20 to 46)	<0.001
K2	−162 (−600 to 729)	76 (−5 to 296)	0.546

**Table 3:** AUC analysis results from the first cohort.

	AUC (95% CI)	Cutoff	Sensitivity (95% CI)	Specificity (95% CI)
ASL	0.994 (0.967–1.000)	2.3	100% (73.5–100.0)	93.3% (68.1–99.8)
Corrected rCBV	0.800 (0.591–1.000)	4.9	66.7% (34.9–90.1)	100.0% (78.2–100.0)
rCBF	0.856 (0.670–0.989)	65.3	75.0% (42.8–94.5)	93.3% (68.1–99.8)

**Table 4:** Clinical and demographic characteristics of patients in the second cohort.

	Hemangioblastoma (HB Group)	Non-Hemangioblastoma (NHB Group)
N	18	27
Age, years (median, range)	46.0 (39.0–55.0)	50.5 (10.5–67.3)
Gender (male)	12 (66.7%)	14 (51.9%)
Location		
Cerebellar hemisphere	11 (61.1%)	14 (51.9%)
Vermis/midline	5 (27.8%)	8 (29.6%)
Cerebellar peduncle involvement	2 (11.1%)	5 (18.5%)
Multiple	1 (5.6%)	6 (22.2%)
Lesion morphology		
Predominantly (>80%) solid	3 (16.7%)	17 (63.0%)
Mixed solid and cystic	5 (27.8%)	3 (11.1%)
Cystic (>80%) with enhanced nodule	10 (55.6%)	6 (22.2%)
Necrotic with irregular wall	0 (0.0%)	1 (3.7%)
Volume (mL)	13.5 (9.6–20.5)	14.3 (9.9–14.7)
Cyst type		
Extratumoral cyst	11 (61.1%)	2 (7.4%)
Intratumoral cyst	6 (33.3%)	9 (33.3%)
Solid	1 (5.6%)	16 (59.3%)
Hydrocephalus	10 (55.6%)	15 (55.6%)
Enhancement pattern: homogeneous	13 (72.2%)	6 (22.2%)

**Table 5:** MRI parameter data in the second cohort.

	HB Group	NHB Group	*p* Value
N	18	27	
Relative ASL median (25–75th percentile)	9.5 (5.4–11.1)	1.2 (0.9–1.5)	<0.001

**Table 6:** **Validation of the fixed relative ASL cutoff (2.3) in cohort 2**.

	N (HB/NHB)	Cutoff (Relative ASL)	Sensitivity (95% CI)	Specificity (95% CI)
Non-overlapping subset of cohort 2 (excluding cohort 1)	18 (6/12)	2.3 (fixed)	100% (54.1–100.0)	91.7% (61.5–99.8)

95% CIs were calculated using the exact binomial (Clopper–Pearson) method.

## Data Availability

The data that support the findings of this study are available from the corresponding author upon reasonable request. The data are not publicly available due to privacy and ethical restrictions.

## Supplementary Materials

The following supporting information can be downloaded at: https://www.mdpi.com/article/10.3390/cancers18121926/s1. Figure S1: Representative hemangioblastoma showing marked hyperperfusion on perfusion MRI; Figure S2: Representative metastatic tumor on perfusion MRI; Figure S3: Distribution of relative ASL values across non-hemangioblastoma (NHB) pathological subtypes in cohort 2; Table S1: Comparison of discrimination among conventional MRI morphology, ASL, combined MRI + ASL, and DSC-based models in cohort 1 with internal validation; Text S1: TRIPOD+AI checklist [26,35,36,37].

## Abbreviations

The following abbreviations are used in this manuscript:

ASL	arterial spin labeling
AUCs	areas under the ROC curve
CIs	confidence intervals
DSC	dynamic susceptibility contrast
FOV	field of view
MRI	magnetic resonance imaging
ROC	receiver operating characteristic
ROI	regions of interest
rCBF	regional cerebral blood flow
rCBV	regional cerebral blood volume
rTBF	relative tumor blood flow
SE-EPI	spin-echo echo-planar imaging
TE	echo time
TR	repetition time

## References

[1] Yin X., Duan H., Yi Z., Li C., Lu R., Li L. (2020). Incidence, prognostic factors and survival for hemangioblastoma of the central nervous system: Analysis based on the surveillance, epidemiology, and end results database. Front. Oncol..

[2] Matsusue E., Inoue C., Tabuchi S., Yoshioka H., Nagao Y., Matsumoto K., Nakamura K., Fujii S. (2022). Advanced magnetic resonance imaging findings of cerebellar hemangioblastomas: A report of three cases and a literature review. Acta Radiol. Open.

[3] Lonser R.R., Glenn G.M., Walther M., Chew E.Y., Libutti S.K., Linehan W.M., Oldfield E.H. (2003). Von Hippel–Lindau disease. Lancet.

[4] Vortmeyer A.O., Falke E.A., Gläsker S., Li J., Oldfield E.H. (2013). Nervous system involvement in von Hippel–Lindau disease: Pathology and mechanisms. Acta Neuropathol..

[5] Yousef A., Rutkowski M.J., Yalcin C.E., Eren O.C., Caliskan I., Tihan T. (2019). Sporadic and Von-Hippel Lindau disease-associated spinal hemangioblastomas: Institutional experience on their similarities and differences. J. Neurooncol..

[6] Hussein M.R. (2007). Central nervous system capillary haemangioblastoma: The pathologist’s viewpoint. Int. J. Exp. Pathol..

[7] Hasselblatt M., Jeibmann A., Gerß J., Behrens C., Rama B., Wassmann H., Paulus W. (2005). Cellular and reticular variants of haemangioblastoma revisited: A clinicopathologic study of 88 cases. Neuropathol. Appl. Neurobiol..

[8] Kim E.H., Moon J.H., Kang S.G., Lee K.S., Chang J.H. (2020). Diagnostic challenges of posterior fossa hemangioblastomas: Refining current radiological classification scheme. Sci. Rep..

[9] Kikuchi K., Hiwatashi A., Togao O., Yamashita K., Kamei R., Yoshimoto K., Iihara K., Suzuki S.O., Iwaki T., Suzuki Y. (2018). Arterial spin-labeling is useful for the diagnosis of residual or recurrent meningiomas. Eur. Radiol..

[10] Sheng Y., Zhao B., Cheng H., Yu Y., Wang W., Yang Y., Ding Y., Qiu L., Qin Z., Yao Z. (2024). A convolutional neural network model for distinguishing hemangioblastomas from other cerebellar-and-brainstem tumors using contrast-enhanced MRI. J. Magn. Reson. Imaging.

[11] Nabavizadeh S.A., Wolf R.L. (2023). Clinical applications of MR perfusion imaging. Functional Neuroradiology.

[12] Cha S., Knopp E.A., Johnson G., Wetzel S.G., Litt A.W., Zigzags D. (2002). Intracranial mass lesions: Dynamic contrast-enhanced susceptibility-weighted echo-planar perfusion MR imaging. Radiology.

[13] Shiroishi M.S., Jones J.G.A., Muradyan N., Lacerda S., Chen B.T., Go J.L., Law M. (2014). MR perfusion imaging: ASL, T2*-Weighted DSC, and T1-Weighted DCE Methods. Functional Brain Tumor Imaging.

[14] Welker K., Boxerman J., Kalnin A., Kaufmann T., Shiroishi M., Wintermark M. (2015). ASFNR recommendations for clinical performance of MR dynamic susceptibility contrast perfusion imaging of the brain. AJNR Am. J. Neuroradiol..

[15] Aronen H.J., Gazit I.E., Louis D.N., Buchbinder B.R., Pardo F.S., Weisskoff R.M., Harsh G.R., Cosgrove G.R., Halpern E.F., Hochberg F.H. (1994). Cerebral blood volume maps of gliomas: Comparison with tumor grade and histologic findings. Radiology.

[16] Knopp E.A., Cha S., Johnson G., Mazumdar A., Golfinos J.G., Zagzag D., Miller D.C., Kelly P.J., Kricheff I.I. (1999). Glial neoplasms: Dynamic contrast-enhanced T2*-weighted MR Imaging. Radiology.

[17] Sugahara T., Korogi Y., Kochi M., Ikushima I., Hirai T., Okuda T., Shigematsu Y., Liang L., Ge Y., Ushio Y. (1998). Correlation of MR imaging-determined cerebral blood volume maps with histologic and angiographic determination of vascularity of gliomas. AJR Am. J. Roentgenol..

[18] Sugahara T., Korogi Y., Shigematsu Y., Liang L., Yoshizumi K., Kitajima M., Takahashi M. (1999). Value of dynamic susceptibility contrast magnetic resonance imaging in the evaluation of intracranial tumors. Top. Magn. Reson. Imaging.

[19] Hussain N.S., Moisi M.D., Keogh B., McCullough B.J., Rostad S., Newell D., Gwinn R., Foltz G., Mayberg M., Aguedan B. (2017). Dynamic susceptibility contrast and dynamic contrast-enhanced MRI characteristics to distinguish microcystic meningiomas from traditional Grade I meningiomas and high-grade gliomas. J. Neurol. Surg..

[20] She D., Yang X., Xing Z., Cao D. (2016). Differentiating hemangioblastomas from brain metastases using diffusion-weighted imaging and dynamic susceptibility contrast-enhanced perfusion-weighted MR imaging. AJNR Am. J. Neuroradiol..

[21] Pons-Escoda A., Garcia-Ruiz A., Garcia-Hidalgo C., Gil-Solsona R., Naval-Baudin P., Martin-Noguerol T., Fernandez-Coello A., Flores-Casaperalta S., Fernandez-Viñas M., Gago-Ferrero P. (2023). MR dynamic-susceptibility-contrast perfusion metrics in the presurgical discrimination of adult solitary intra-axial cerebellar tumors. Eur. Radiol..

[22] Kumar V.A., Knopp E.A., Zagzag D. (2010). Magnetic resonance dynamic susceptibility-weighted contrast-enhanced perfusion imaging in the diagnosis of posterior fossa hemangioblastomas and pilocytic astrocytomas: Initial results. J. Comput. Assist. Tomogr..

[23] Kurokawa R., Kurokawa M., Baba A., Kim J., Capizzano A., Bapuraj J., Srinivasan A., Moritani T. (2022). Differentiation of pilocytic astrocytoma, medulloblastoma, and hemangioblastoma on diffusion-weighted and dynamic susceptibility contrast perfusion MRI. Medicine.

[24] Vella S., Lauri J., Grech R. (2025). Comparison of arterial spin-labeling and DSC perfusion MR imaging in pediatric brain tumors: A systematic review and meta-analysis. AJNR Am. J. Neuroradiol..

[25] Boxerman J.L., Schmainda K.M., Weisskoff R.M. (2006). Relative cerebral blood volume maps corrected for contrast agent extravasation significantly correlate with glioma tumor grade, whereas uncorrected maps do not. AJNR Am. J. Neuroradiol..

[26] Collins G.S., Moons K.G.M., Dhiman P., Riley R.D., Beam A.L., Van Calster B., Ghassemi M., Liu X., Reitsma J.B., van Smeden M. (2024). TRIPOD+AI statement: Updated guidance for reporting clinical prediction models that use regression or machine learning methods. BMJ.

[27] Noguchi T., Yoshiura T., Hiwatashi A., Togao O., Yamashita K., Nagao E., Shono T., Mizoguchi M., Nagata S., Sasaki T. (2008). Perfusion imaging of brain tumors using arterial spin-labeling: Correlation with histopathologic vascular density. AJNR Am. J. Neuroradiol..

[28] Kang K.M., Sohn C.H., You S.H., Nam J.G., Choi S.H., Yun T.J., Yoo R.E., Kim J.H. (2017). Added value of arterial spin-labeling MR imaging for the differentiation of cerebellar hemangioblastoma from metastasis. AJNR Am. J. Neuroradiol..

[29] Yamashita K., Yoshiura T., Hiwatashi A., Togao O., Yoshimoto K., Suzuki S.O., Kikuchi K., Mizoguchi M., Iwaki T., Honda H. (2012). Arterial spin labeling of hemangioblastoma: Differentiation from metastatic brain tumors based on quantitative blood flow measurement. Neuroradiology.

[30] Ata E.S., Turgut M., Eraslan C., Dayanır Y.Ö. (2016). Comparison between dynamic susceptibility contrast magnetic resonance imaging and arterial spin labeling techniques in distinguishing malignant from benign brain tumors. Eur. J. Radiol..

[31] Ye J., Bhagat S.K., Li H., Luo X., Wang B., Liu L., Yang G. (2016). Differentiation between recurrent gliomas and radiation necrosis using arterial spin labeling perfusion imaging. Exp. Ther. Med..

[32] Maral H., Ertekin E., Tunçyürek Ö., Özsunar Y. (2020). Effects of Susceptibility Artifacts on Perfusion MRI in Patients with Primary Brain Tumor: A Comparison of arterial Spin-Labeling versus DSC. AJNR Am. J. Neuroradiol..

[33] Simsek O., Sheth N., Manteghinejad A., Wannasarnmetha M., Roberts T.P., Bhatia A. (2024). Arterial spin-labeling perfusion lightbulb sign: An imaging biomarker of pediatric posterior fossa hemangioblastoma. AJNR Am. J. Neuroradiol..

[34] Athisayamani S., Singh A.R., Joshi G.P., Cho W. (2025). Three-Stage Transfer Learning with AlexNet50 for MRI Image Mul-ti-Class Classification with Optimal Learning Rate. Comput. Model. Eng. Sci..

[35] Harrell F.E., Lee K.L., Mark D.B. (1996). Multivariable prognostic models: Issues in developing models, evaluating assumptions and adequacy, and measuring and reducing errors. Stat. Med..

[36] Morimoto S. makedatar: Load and Prepare Clinical Trial Data from Column-Info Excel Workbooks (Version 0.1.0). GitHub Repository, 2026. https://github.com/Shimpeim/makedatar.

[37] DeLong E.R., DeLong D.M., Clarke-Pearson D.L. (1988). Comparing the areas under two or more correlated receiver operating characteristic curves: A nonparametric approach. Biometrics.

