# Supplementary material for: Arterial Spin Labeling Magnetic Resonance Imaging Can Identify Posterior Fossa Hemangioblastoma: Comparison with Dynamic Susceptibility Contrast
Source: Cancers (Basel). 2026 Jun 12;18(12):1926. doi: 10.3390/cancers18121926 (PMC13296456; doi:10.3390/cancers18121926)
Supplement: Supplementary file 1 [file cancers-18-01926-s001.zip › cancers-4296715-supplementary-2.pdf]

cancers

MDPI

Supplementary Material: Arterial Spin Labeling Magnetic Resonance Imaging Can Identify Posterior Fossa Hemangioblastoma: Comparison with Dynamic Susceptibility Contrast

Takeshi Hiu, Ayano Ishiyama, Minoru Morikawa, Shimpei Morimoto, Ayaka Matsuo, Hikaru Nakamura, Hirofumi Koike, Yaojing Lin, Shiro Baba, Kenta Ujifuku, Koichi Yoshida, Ryo Toya and Takayuki Matsuo

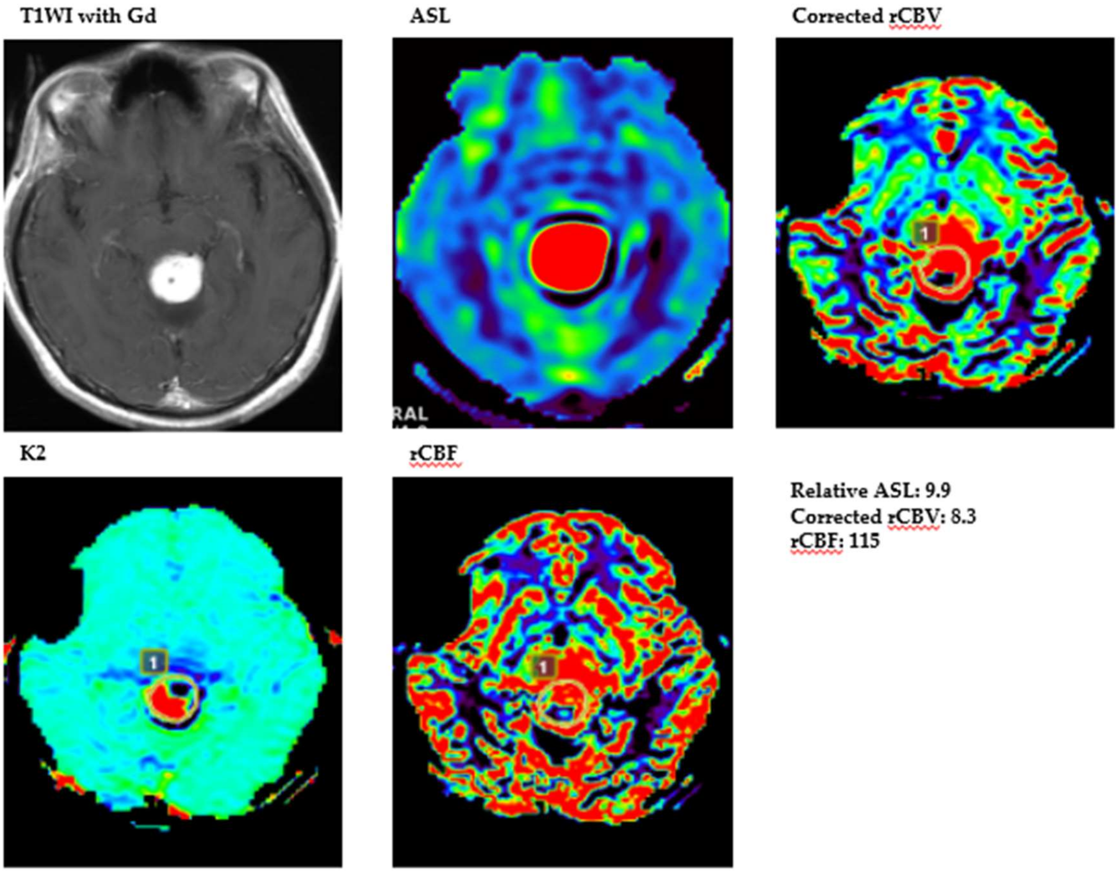

Figure S1. Representative hemangioblastoma showing marked hyperperfusion on perfusion MRI. Contrast-enhanced T1-weighted image (T1WI with Gd) demonstrates an avidly enhancing solid component. Corresponding perfusion maps show pronounced hyperperfusion on ASL and elevated DSC-derived parameters (corrected rCBV and rCBF) with permeability information (K2). Relative ASL, corrected rCBV (unitless), and rCBF (mL/100 mL/min) values measured within the solid enhancing component are shown.

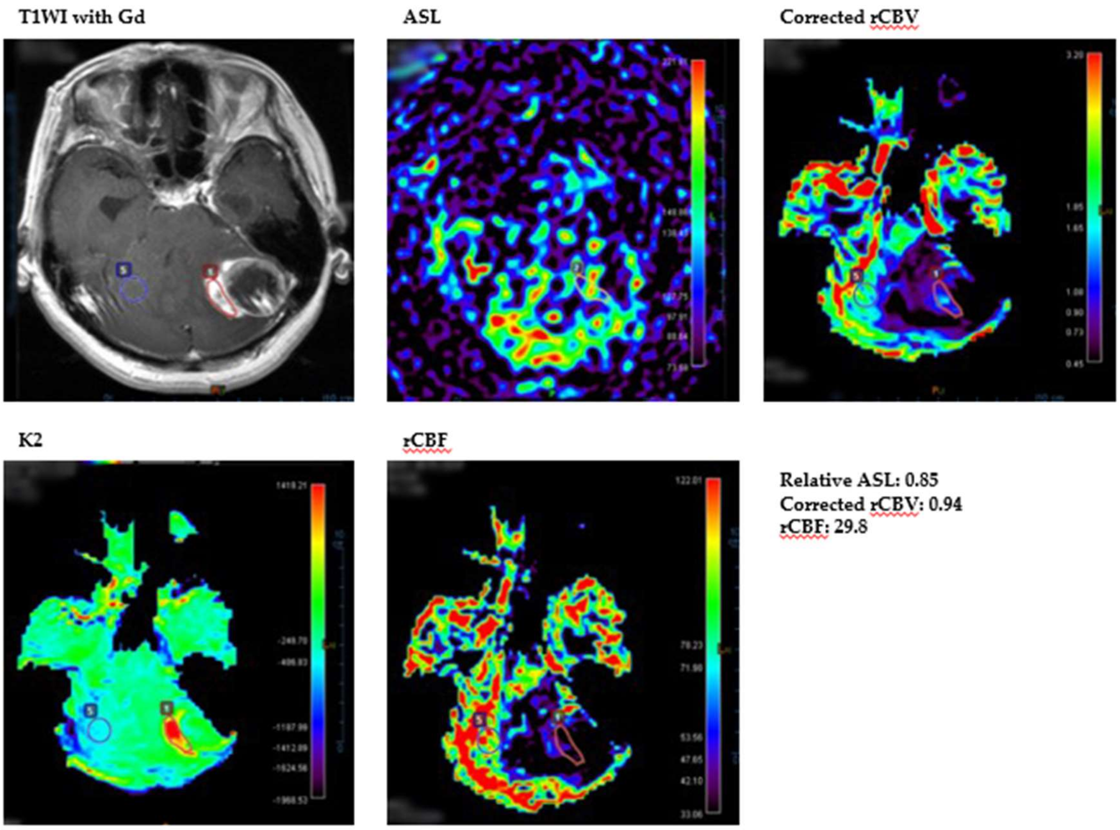

Figure S2. Representative metastatic tumor on perfusion MRI. Contrast-enhanced T1-weighted image (T1WI with Gd) and corresponding perfusion maps (ASL, corrected rCBV, rCBF, and K2) are shown. Relative ASL, corrected rCBV (unitless), and rCBF (mL/100 mL/min) values measured within the enhancing tumor component are provided.

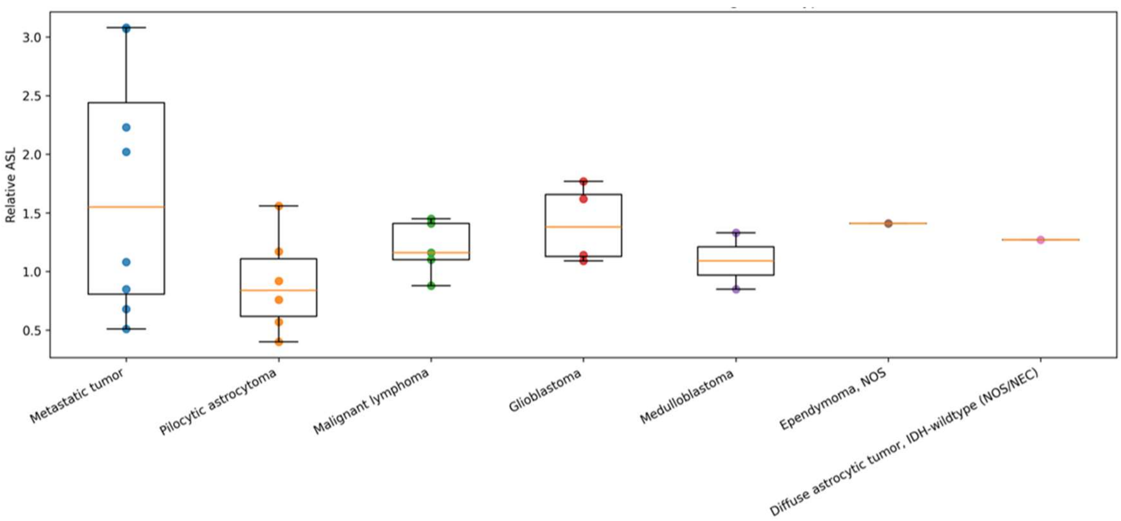

Figure S3. Distribution of relative ASL values across non-hemangioblastoma (NHB) pathological subtypes in cohort 2. Boxplots with overlaid individual data points are shown for each NHB subtype. Values are presented descriptively (median and range), and no formal statistical testing was performed due to small sample sizes within subtypes.

Table S1. Comparison of discrimination among conventional MRI morphology, ASL, combined MRI+ASL, and DSC-based models in cohort 1 with internal validation. Logistic regression models were fitted in cohort 1 using pre-specified predictors: MRI-only (enhancing mural nodule present, extratumoral cyst present, homogeneous enhancement), ASL-only (relative ASL), MRI+ASL (MRI-only predictors + relative ASL), and DSC model (corrected rCBV and rCBF). Discrimination was summarized by AUC with 95% CIs (DeLong), and AUCs were compared using the paired DeLong test (p values shown vs MRI-only). Internal validation was performed using bootstrap resampling (B = 2000; seed = 20260513) and the Harrell optimism-correction approach to report optimism-corrected AUCs.

	Predictors	AUC (apparent)	AUC (bootstrap 95% CI)	DeLong p vs MRI-only	Optimism-corrected AUC (B = 2000)
**MRI-only**	mural nodule + extratumoral cyst + homogeneous enhancement	0.786	0.647–0.789	—	0.726
**ASL-only**	Relative ASL	0.994	0.994–0.994	0.021	0.994
**MRI + ASL**	mural nodule + extratumoral cyst + homogeneous enhancement + relative ASL	1.000	0.856–1.000	0.015	0.990
**DSC model**	corrected rCBV + rCBF	0.833	0.694–0.856	0.729	0.800

Footnotes:

- AUCs and 95% CIs were estimated using the DeLong method; AUCs were compared using the paired DeLong test (p values shown vs. MRI-only).
- Internal validation used bootstrap resampling (B = 2000; seed = 20260513) with the Harrell optimism-correction approach to obtain optimism-corrected AUCs.

Table S1.

The item numbers in this report correspond with TRIPOD+AI [26] item numbering.

Abstract (Item 2)

Background and objectives: The clinical and scientific background should be referred to in the main text. The objective of the analyses reported in this document was to assess the diagnostic performance of the primary marker compared with that of the comparator marker for distinguishing hemangioblastoma from other intracranial diagnoses, using ROC-based analysis with internal and external validation. **Methods:** Diagnostic accuracy was evaluated in a development cohort (see Section 2.1 in the main text, n = 27) and an external validation cohort (see Section 2.1 in the main text, n = 18). ROC curves were constructed for each marker; the AUC with 95% confidence intervals (CI) was estimated by the DeLong method, and the two AUCs were compared using the paired DeLong test. The optimal diagnostic threshold was selected by maximizing the Youden index (sensitivity + specificity − 1). Internal validation used the Harrell bootstrap optimism correction [35] (B = 2000 replicates). **Results:** In the development cohort, the AUC for the primary marker (relative ASL) was 0.994 (95% CI, 0.979–1.000) and that for the comparator marker (corrected rCBV) was 0.800 (95% CI, 0.581–1.000) (DeLong comparison: p = 0.072). In the external validation cohort, the AUC for the primary marker was 1.000 (95% CI, 1.000–

1.000), and the AUC for the comparator marker was not available due to the study design (see Section 2.1). **Conclusion:** Relative ASL showed superior diagnostic performance compared with corrected rCBV for identifying posterior fossa hemangioblastoma, and the fixed cutoff of 2.3 demonstrated high sensitivity and specificity in a non-overlapping validation subset; independent multi-center validation is warranted.

1. Introduction

1.1. Background and Rationale (TRIPOD+AI Item 3a)

The clinical problem, condition of interest (hemangioblastoma), and existing evidence on MRI perfusion markers, as well as the rationale for comparing relative ASL and corrected rCBV, are summarized in the main text.

1.2. Target Population and Intended Purpose (TRIPOD+AI Item 3b)

The target population for which this diagnostic model is intended (patient characteristics, clinical setting, stage of the care pathway) is described in the Introduction section in the main text.

1.3. Health Inequalities (TRIPOD+AI Item 3c)

Health inequality between sociodemographic groups was not evaluated because the study collected only clinical data.

1.4. Study Objectives (TRIPOD+AI Item 4)

The objectives of this study were:

1) To assess the diagnostic performance of the primary marker, relative ASL (variable name: Rel_ASL) and comparator marker, corrected rCBV (variable name: rCBV_corrected), for distinguishing **hemangioblastoma** from other intracranial diagnoses.
2) To compare the AUC of the two markers using the DeLong method.
3) To validate performance internally (bootstrap optimism correction) and externally (independent cohort).

2. Methods

2.1. Data Sources (TRIPOD+AI Items 5a–5b)

The data sources for both the development and validation cohorts, along with their rationales and data collection period, are described in Section 2 of the main text.

2.2. Study Setting (TRIPOD+AI Item 6a)

- See Section 2.1 in the main text for the clinical institution and department providing the data and the data collection period.
- See Sections 2.1 and 2.2 for the clinical context in which MRI perfusion imaging was performed and for the technological details of the procedure.
- See Section 2.1 in the main text for the data source information.

2.3. Participants (TRIPOD+AI Items 6b–6c)

The eligibility criteria applied to each cohort are described in Section 2.1 in the main text.

The development cohort comprised **27 participants** (12 with hemangioblastoma,15 with other diagnoses). The validation cohort comprised **18 participants** (6 with hemangioblastoma, 12 with other diagnoses).

2.4. Data Pre-processing and Quality Checking (TRIPOD+AI Item 7)

https://doi.org/10.3390/cancers18121926

All data pre-processing and quality checking steps applied before analysis are described in Section 2.3 in the main text.

2.5. Outcome (TRIPOD+AI Items 8a–8c)

2.5.1. Outcome definition (Item 8a)

The reference standard was the clinical or histopathological diagnosis recorded in the variable diagnosis. The binary outcome was defined as hemangioblastoma (positive class, coded 1) versus all other diagnoses (negative class, coded 0), using the labelled factor encoding produced by the makedatar data pipeline [36].

2.5.2. Outcome assessors (Item 8b)

The reference standard (diagnosis) was determined by histopathological examination of surgical specimens in routine clinical practice at our institution. Diagnoses were rendered by board-certified pathologists/neuropathologists in the Department of Pathology, and patients without histopathological confirmation were excluded.

2.5.3. Blinding of outcome assessment (Item 8c)

Blinding of outcome assessment (Item 8c): Histopathological diagnoses were established as part of routine clinical care and were therefore independent of the quantitative perfusion marker values used in this study. The radiologists/neurosurgeon readers who performed ROI delineation and extracted perfusion metrics were blinded to the final histopathological diagnosis at the time of ROI placement.

2.6. Predictors (TRIPOD+AI Items 9a–9c)

2.6.1. Choice and pre-selection of predictors (Item 9a)

The rationale for selecting relative ASL and corrected rCBV as the markers under evaluation is stated in the Introduction section in the main text. Variable selection using a machine learning method was not performed in this study.

2.6.2. Predictor definitions (Item 9b)

Two quantitative MRI perfusion markers were evaluated:

Relative ASL (variable name: Rel_ASL) — primary marker.

Corrected rCBV (variable name: rCBV_corrected) — comparator marker.

Details of the measurement method (MRI sequence, software, and ROI placement strategy), units of measurement, any pre-processing, and when and how the marker was measured related to the diagnosis are described in Section 2.3.

2.6.3. Predictor assessors (Item 9c)

Manual ROI delineation, computation methods, and standardizations are described in Section 2.3 in the main text.

2.7. Sample Size (TRIPOD+AI Item 10)

No formal sample size calculation was performed; the analysis used all available participants in each cohort (Cohort 1: n = 27; Cohort 2: n = 18). Observed numbers are reported in Section 5.1.

2.8. Missing Data (TRIPOD+AI Item 11)

No missing values were present in the outcome variable or the index test variables. Therefore, no exclusion or imputation was required.

https://doi.org/10.3390/cancers18121926

In the external validation cohort (Cohort 2), the following marker was not measured and is therefore absent from the validation performance analysis: **corrected rCBV**.

Performance metrics for available markers are reported from the subset of participants with non-missing values. Consequently, all analyses reported in the main text were the same data set within each of the development and validation cohort.

2.9. Analytical Methods (TRIPOD+AI Item 12)

2.9.1. Data usage and partitioning (Item 12a)

Data were not randomly partitioned. The development cohort (Cohort 1) and external validation cohort (Cohort 2) were defined a priori by data source. No additional held-out test set was created from the development data; internal validation was performed by bootstrap resampling (see Section 2.9.3).

2.9.2. Predictor handling (Item 12b)

The predictors were used on their original scale. Transformation was not applied.

2.9.3. Model type, building steps, and internal validation (Item 12c)

ROC curves were constructed for Rel_ASL and rCBV_corrected separately. The AUC with 95% CI was estimated using the DeLong method [37]. The two AUCs were compared using the paired DeLong test[4]. The optimal diagnostic threshold for each marker was selected by **maximizing the Youden index (sensitivity + specificity − 1)**. Sensitivity (Se), specificity (Sp), positive predictive value (PPV), and negative predictive value (NPV) at the optimal threshold were calculated with exact 95% CIs (Clopper–Pearson method).

Bootstrap internal validation used B = **2000** replicates (random seed: 20260513) following the Harrell optimism correction approach[2]. For each bootstrap replicate: (i) the optimal threshold was identified on the bootstrap sample (apparent performance); (ii) that threshold was applied to the original development data (test performance); (iii) optimism was computed as the difference; (iv) bias-corrected (BC) estimates were obtained by subtracting mean optimism from the apparent development estimates. Bootstrap percentile 95% CIs for Se, Sp, PPV, and NPV were also derived.

2.9.4. Heterogeneity across clusters (Item 12d)

No pre-specified subgroup analyses were planned. Stratification by additional clinical or demographic variables was not performed.

2.9.5. Performance measures and plots (Item 12e)

Model performance was summarized by AUC (discrimination) and Se, Sp, PPV, and NPV at the optimal threshold (classification performance). ROC curves were plotted for each marker. CIs used the DeLong method for AUC and Clopper–Pearson exact method for proportions.

2.9.6. Model recalibration and updating (Item 12f)

Model recalibration or updating was not performed. The development-fitted threshold was applied unchanged to the validation cohort.

2.9.7. Prediction calculation for evaluation (Item 12g)

For external validation, the threshold identified in the development cohort was applied without modification to the validation cohort. All continuous marker values were used directly; no rescaling was applied at the evaluation stage.

All analyses were performed in **R version 4.4.1 (2024-06-14)** using the **pROC** package version 1.18.5.

2.10. Class Imbalance (TRIPOD+AI Item 13)

The positive: negative ratio in the development cohort was considered balanced. Class imbalance methods were not used.

2.11. Fairness (TRIPOD+AI Item 14)

Because the study design did not include sociodemographic information beyond sex, formal fairness analysis was not conducted. The sex balance was well-balanced (see Tables 1 and 4 in the main text). It is assumed that all participants were categorized as of Eastern Asian origin and having a culturally conventional Japanese lifestyle, based on the demographic characteristics of patients consulting the institute where the data were collected.

2.12. Model Output (TRIPOD+AI Item 15)

The model outputs a continuous score for each marker. Classification was performed by applying a fixed threshold, identified in the development cohort by **Youden index**. Scores above the threshold are classified as **hemangioblastoma** (positive); scores at or below it are classified as negative.

2.13. Differences Between Development and Evaluation Data (TRIPOD+AI Item 16)

The MRI protocol and image analysis were detailed in Sections 2.2 and 2.3 in the main text.

2.14. Ethical Approval (TRIPOD+AI Item 17)

Section 2.4 and the footnote sections (**Institutional Review Board Statement** and **Informed Consent Statement**) in the main text name the ethics committee that approved this study and address the informed consent procedures.

3. Open Science (TRIPOD+AI Item 18)

3.1. Funding (Item 18a)

All sources of funding for this study are declared in the footnote section (**Fundings**) in the main text. The funder did not participate in the data collection and generation, analysis, and interpretations of data and the result from analyses.

3.2. Conflicts of Interest (Item 18b)

There is no conflict of interest relevant to the results and conclusions, including discussions during the study design, plan, analysis, and interpretation of the results.

3.3. Protocol (Item 18c)

There is no study protocol publication including on a public repository. The study protocol, written in Japanese, is available from the corresponding author upon reasonable request.

3.4. Study Registration (Item 18d)

There is no registration of this study on a public repository, aligning with the conventional flow of clinical research within the institute to which the author was affiliated.

3.5. Data Availability (Item 18e)

https://doi.org/10.3390/cancers18121926

The data that support the findings of this study are available from the corresponding author upon reasonable request. The data are not publicly available due to privacy and ethical restrictions.

3.6. Analytical Code Availability (Item 18f)

The analytical code used in developing and evaluating the predictive model will be uploaded to a GitHub repository after publication of the main text. The makedatar pipeline [36] is available at [https://github.com/Shimpeim/makedatar].

4. Patient and Public Involvement (TRIPOD+AI Item 19)

Any patient and public involvement activity was not included during the design, conduct, reporting, interpretation, or dissemination of this study.

5. Results

5.1. Participants (TRIPOD+AI Items 20a–20c)

5.1.1. Participant flow (Item 20a)

Cohort	Role	Total n	Hemangioblastoma (positive)	Other (negative)
Development cohort	Development	27	12	15
Validation cohort	External validation	18	6	12

5.1.2. Participant characteristics (Item 20b)

See Tables 1 and 4 for age, sex, and marker distributions by cohort and by outcome class.

5.1.3. Development versus evaluation comparison (Item 20c)

The distribution of key predictors (Rel_ASL, rCBV_corrected) is summarized below for each cohort. Other key participant characteristics in the development and validation cohorts are described in the main text (Tables 1 and 4). Any clinically meaningful differences that may affect the generalizability of the performance estimates are highlighted.

Participant characteristics in the Development cohort

Primary predictor (Relative ASL)

Statistics	Hemangioblastoma (n = 12)	Other (n = 15)
n (non-missing)	12	15
Mean (SD)	8.97 (4.22)	1.34 (0.681)
Median [Q1–Q3]	9.62 [5.18–11.6]	1.16 [0.887–1.59]
Min–Max	2.46–16.8	0.512–3.08

Comparator predictor (corrected rCBV)

Statistics	Hemangioblastoma (n = 12)	Other (n = 15)
n (non-missing)	12	15
Mean (SD)	5 (3.25)	1.36 (1.06)
Median [Q1–Q3]	5.75 [1.99–7.18]	0.95 [0.71–1.81]
Min–Max	0.08–10	0.29–4.47

Participant characteristics in the Validation cohort

Primary predictor (Relative ASL)

Statistics	Hemangioblastoma (n = 6)	Other (n = 12)
n (non-missing)	6	12
Mean (SD)	9.09 (5.41)	1.27 (0.683)
Median [Q1–Q3]	8.27 [5.88–10]	1.18 [0.875–1.48]
Min–Max	3.43–18.9	0.401–3.07

Comparator predictor (corrected rCBV)

Statistics	Hemangioblastoma (n = 6)	Other (n = 12)
n (non-missing)	0	0
Mean (SD)	NA	NA
Median [Q1–Q3]	NA	NA
Min–Max	NA	NA

5.2. Participants and Events per Analysis (TRIPOD+AI Item 21)

Analysis	Cohort	Total n	Events (Hemangioblastoma)	Nonevents
ROC development	Development cohort	27	12	15
Bootstrap internal validation	Development cohort	27	12	15
External validation	Validation cohort	18	6	12

Each bootstrap sample has the same n as the development cohort (B = 2000 resamples with replacement).

5.3. Model Specification (TRIPOD+AI Item 22)

This analysis evaluates individual continuous markers without a composite scoring model; no regression coefficients require reporting. The threshold values identified in the development cohort (reported in Section 5.4) are sufficient for classification in new individuals.

5.4. Diagnostic Performance (TRIPOD+AI Items 23a–23b)

AUC 95% CI: DeLong method. Se, Sp, PPV, NPV 95% CI: Clopper–Pearson exact method. BC = bias-corrected (bootstrap optimism correction, B = 2000). Cutoffs derived in the development cohort using **maximizing the Youden index (sensitivity + specificity − 1)**.

Stratum: Overall

Development cohort — n = 27 (12 Hemangioblastoma, 15 other)

Marker	AUC (95% CI)	Cutoff	Se (95% CI)	Sp (95% CI)	PPV (95% CI)	NPV (95% CI)
Primary	0.99 (0.98–1.0)	2.3	100% (74–100%)	93.3% (68.1–99.8%)	92.3% (64.0–99.8%)	100% (76.8–100%)
Comparator	0.80 (0.58–1.0)	4.9	66.7% (34.9–90.1%)	100% (78.2–100%)	100% (63.1–100%)	78.947% (54.435–93.948%)

DeLong comparison (Rel_ASL vs rCBV_corrected): p = 0.072.

Internal validation (B = 2000, Harrell optimism correction)

Marker	Apparent AUC	BC AUC	Optimism	BC Se (Bootstrap 95% CI)	BC Sp (Bootstrap 95% CI)	BC PPV (Bootstrap 95% CI)
Primary	0.99	0.994	0.000	96.8% (100.0–100.0%)	91.7% (78.6–100.0%)	90.7% (75.0–100.0%)
Comparator	0.80	0.801	-0.001	62.8% (37.5–91.7%)	95.4% (100–100%)	94.8% (100–100%)

External validation cohort (Cohort 2) — n = 18 (6 Hemangioblastoma, 12 other)

Marker	AUC (95% CI)	Cutoff applied	Se (95% CI)	Sp (95% CI)	PPV (95% CI)	NPV (95% CI)
Primary	1.0 (1.0–1.0)	2.3 (fixed)	100.0% (54.1–100.0%)	91.7% (61.5–99.8%)	85.7% (42.1–99.6%)	100.0% (71.5–100.0%)
Comparator	*not measured in the validation cohort*	—	—	—	—	—

5.5. Model Updating (TRIPOD+AI Item 24)

Model updating or recalibration was not performed. The development-fitted model and threshold were applied without modification to the validation cohort.

https://doi.org/10.3390/cancers18121926

6. Discussion

6.1. Interpretation (TRIPOD+AI Item 25)

- The relative diagnostic performance of relative ASL versus corrected rCBV showed that relative ASL was superior to corrected rCBV.
- The clinical significance of the optimal cutoff values was not evaluated because the predictors are instrumental parameters rather than functional clinical evaluations.
- The performance observed in external validation supports the intended clinical purpose; however, the small sample size of participants and the homogeneities in data generation (participants, assessors and diagnosis from a single institution across the development and validation cohort) should be considered (see Sections 2.1, 2.2, and 2.3 in the main text).

6.2. Limitations (TRIPOD+AI Item 26)

- Retrospective design; potential for latent selection and information bias.
- Fairness and performance equity across sociodemographic groups were not formally assessed.
- Other limitations arising from the study design are described in Section 4 of the main text.

6.3. Usability (TRIPOD+AI Item 27)

6.3.1. Handling poor quality or unavailable input data (Item 27a)

The comparison of predictors was the objective of this study, which did not include assessments that would require a resource for defining usability.

6.3.2. User requirements (Item 27b)

The comparison of predictors was the objective of this study, which did not include assessments that would require a resource for defining usability.

6.3.3. Next steps for research (Item 27c)

The Discussion section includes the limitations caused by the study design and improvement, which would be assessed in a future study.

https://doi.org/10.3390/cancers18121926
